# High genetic diversity in the *Culex pipiens* complex from a West Nile Virus epidemic area in Southern Europe

**DOI:** 10.1186/s13071-016-1429-1

**Published:** 2016-03-15

**Authors:** Mauro Simonato, Isabel Martinez-Sañudo, Giacomo Cavaletto, Giacomo Santoiemma, Andrea Saltarin, Luca Mazzon

**Affiliations:** Department of Agronomy, Food, Natural Resources, Animals, & Environment (DAFNAE), University of Padua, Viale dell’Università 16, 35020 Legnaro, PD Italy; Istituto Tecnico Agrario “O. Munerati”, Via Capello 10, 45010 Sant’Apollinare, RO Italy

**Keywords:** *Culex pipiens* complex, Mosquitoes, Mitochondrial DNA, Cytochrome oxidase, Nuclear DNA, Acetylcholinesterase-2, Genetic diversity, West Nile Virus

## Abstract

**Background:**

The *Culex pipiens* complex includes the most widespread mosquito species in the world. *Cx. pipiens* is the primary vector of the West Nile Virus (WNV) in Europe and North America. Cases of WNV have been recorded in Italy since 1998. In particular, wet areas along the Po River are considered some of the most WNV affected areas in Italy. Here, we analyzed the genetic structure of ten *Cx. pipiens* populations collected in the last part of the Po River including the Delta area.

**Methods:**

We assessed the genetic variability of two mitochondrial markers, cytochrome oxidase 1 (COI) and 2 (COII), for a total of 1200 bp, and one nuclear marker, a fragment of acetylcholinesterase-2 (ace-2), 502 bp long. The effect of the landscape features was evaluated comparing haplotype and nucleotide diversity with the landscape composition.

**Results:**

The analysis showed a high genetic diversity in both COI and COII gene fragments mainly shared by the populations in the Delta area. The COI-COII network showed that the set of haplotypes found was grouped into three main supported lineages with the higher genetic variability gathered in two of the three lineages. By contrast, ace-2 fragment did not show the same differentiation, displaying alleles grouped in a single clade. Finally, a positive correlation between mitochondrial diversity and natural wetland areas was found.

**Conclusions:**

The high mitochondrial genetic diversity found in *Cx. pipiens* populations from the Po River Delta contrasts with the low variability of inland populations. The different patterns of genetic diversity found comparing mitochondrial and nuclear markers could be explained by factors such as differences in effective population size between markers, sex biased dispersal or lower fitness of dispersing females. Moreover, the correlation between genetic diversity and wetland areas is consistent with ecosystem stability and lack of insecticide pressure characteristic of this habitat. The mtDNA polymorphism found in the Po River Delta is even more interesting due to possible linkages between the mitochondrial lineages and different biting behaviors of the mosquitoes influencing their vector ability of arboviral infections.

**Electronic supplementary material:**

The online version of this article (doi:10.1186/s13071-016-1429-1) contains supplementary material, which is available to authorized users.

## Background

The *Culex pipiens* complex includes two of the most widespread mosquito species in the world, *Cx. pipiens* L. (the northern house mosquito) in temperate climates and *Cx. quinquefasciatus* Say (the southern house mosquito) in tropical and subtropical climates. Although still under debate, two Palaearctic forms or biotypes are usually recognized within *Cx. pipiens*, the pipiens and the molestus forms [1, 2]. The two forms are morphologically very similar, nonetheless the two forms exhibit important behavioral and physiological differences, such as host choice and breeding sites [3, 4].

The mosquitoes of the *Cx. pipiens* complex are considered to be the primary vectors of the West Nile Virus (WNV) in Europe and North America [5, 6]. The cycle of infection involves birds and mosquitoes. In particular, migratory birds are instrumental in the introduction of the virus to temperate areas of Eurasia during spring migrations [7]. The mosquitoes belonging to the *Cx. pipiens* complex feed on both avian and mammalian hosts. This behavior assigns to mosquitoes of the complex the role of bridge-vector for the transmission of the WNV to birds and mammals, including humans [5, 8]. In Italy, after the first report in 1998, WNV became endemic in the North-East after 2008 [9, 10] and since then, in this area, it has been constantly detected in humans, in animals and in the vector mosquitoes [11]. In particular, the areas considered the most affected by WNV outbreak are the wet areas surrounding the Po River, in the Veneto and Emilia Romagna regions [12, 13]. Moreover, the recent finding of simultaneous circulation of two WNV lineages in this area confirms North-Eastern Italy as a high-risk area for WNV emergence [14].

This scenario has drawn attention to mosquitoes of the *Cx. pipiens* complex inhabiting the last part of the Po River. In this area, urban zones are mostly alternated with large cultivated areas, rice paddies and natural wetlands. Here, during summer, *Cx. pipiens* achieves high population densities, creating much annoyance. Despite its epidemiological relevance, population genetic data for the *Cx. pipiens* complex in this WNV outbreak area are still lacking. We consider it particularly important to understand the population structure and the distribution patterns of *Cx. pipiens* in a WNV epidemic area. Genetic variability in local *Cx. pipiens* populations could underlie biological differences in biting and flight behavior, and in spatial and temporal distribution, with effects on the epidemiological patterns of the WNV infection.

Up to date, some surveys about genetic variability for *Cx. pipiens* complex have been carried out in several European countries using allozymes and mitochondrial markers [15–17]. These studies showed a low genetic variability in most of the populations analyzed compared to the closely related species *Cx. torrentium* Martini, morphologically very similar to *Cx. pipiens* and occurring in sympatry with this species throughout Europe and some parts of Asia [1]. Other genetic studies focused on the sympatric occurrence of the molestus and pipiens forms, underlining the presence of asymmetric introgression between the two forms and its implications for the WNV transmission [18, 19].

In this study we inferred the genetic structure of the *Cx. pipiens* complex along the last part of the Po River using both mitochondrial and nuclear markers. In detail, we studied the genetic diversity of cytochrome *c* oxidase subunit 1 (COI) and the cytochrome *c* oxidase subunit 2 (COII) in the populations considered. The cytochrome *c* oxidase fragment has been frequently used to study mosquito species complexes as well as to compare phylogeographic patterns within closely related taxa e.g. [20–22] due to the high number of copies per cell of the mitochondrial genome, its maternal inheritance and its rare recombination rate. Moreover, in order to investigate the diversity of *Culex* mosquitoes and to identify possible cryptic taxa, we analyzed the acetylcholinesterase-2 gene (ace-2), a protein-coding gene often used for discrimination within the *Culex* complex species [21, 23, 24]. Finally, we assessed the possible effect of landscape composition on the genetic diversity. Landscape composition is known to influence mosquito density and diversity [25, 26] but, to our knowledge, studies associating genetic variability and landscape composition are still lacking for *Cx. pipiens*. This information could help to better identify landscape features that may affect the population structure of mosquitoes, mainly in a high-risk area for arbovirus outbreaks.

## Methods

### Study area

Mosquito collection was carried out in the Province of Rovigo (Veneto Region-North Eastern Italy), an area of about 1,500 km^2^. The Rovigo Province consists of a plain located between the lower portion of the rivers Adige and Po, the two longest Italian rivers. The study region is a wide lowland area with large zones below sea level and a network of embanked rivers and channels.

The eastern part, facing the Adriatic Sea, terminates in the Po River Delta, which covers an area of about 380 km^2^. The Po Delta area is characterized by intensive agriculture (rice paddies, maize, wheat) due to its flat topography and the abundance of surface waters for irrigation. Moreover, lagoons and wooded wetland patches, with high biodiversity, are frequent.

The Po Delta is an area located at the crossroads of the most important bird migration routes connecting Europe, the Mediterranean Basin and Africa; moreover, several equine stables are present [27–30]. This area, with more than 300 species of birds, is considered the most important ornithological area in Italy and one of the most relevant in Europe. For these reasons, since 1997 the Po Delta wetlands have been protected by the institution of the Regional Po Delta Park.

### Sample collection

Mosquito specimens were collected twice a month during summer and autumn in 2012. A total of ten geo-referenced sampling sites were chosen for insect collection: nine sites located in the Rovigo Province and one, used as an out-group, located in the Venice Province (Table 1).

**Table 1:** Geographic localization of *Culex pipiens* complex populations analyzed and landscape composition percentage

ID	Collection site	Latitude	Longitude	% Urban	% Rural	%Tree crops	%Wetland
S01	San Sisto	45° 2’44.04” N	11°48’2.27” E	45.3	50.3	1.4	3.0
S02	Sant’Apollinare	45° 2’50.02” N	11°49’52.65” E	13.6	79.2	5.5	1.8
S03	Villa Dose	45° 5’57.10” N	11°53’0.44” E	6.3	88.8	0.4	4.4
S04	Beverare	45° 8’40.16” N	11°56’14.76” E	10.9	71.1	10.4	7.6
S05	Adria	45° 2’11.88” N	12° 3’58.30” E	11.8	80.9	2.0	5.3
S06	PortoViro	45°01’51.71” N	12°14’34.40” E	28.4	67.4	0.4	3.8
S07	Valle Sagreda	45° 02’40.49” N	12°19’10.66” E	4.1	54.0	0.0	41.9
S08	Donzella	44°55’35.47” N	12°19’43.13” E	16.0	43.4	8.0	32.6
S09	Po di Gnocca	44°53’44.46” N	12°19’43.00” E	5.7	84.7	2.4	7.3
S10	Mirano (outgroup)	45°29’37.60” N	12°07’26.79” E	48.8	43.6	6.8	0.8

Landscape composition (%) was calculated in a radius of 1,000 m around on the coordinates of the sampling sites

Mosquitoes were collected as larvae or pupae from drainage or natural ditches using the dipper method. At each visit, larvae and pupae were sampled with at least five dips for each collection site, using half a liter of water, at randomly located points taken at about 30 m intervals along the ditches to avoid sampling bias. After collection, the samples were transferred to the laboratory, reared to the adult stage, identified and then stored in 80 % ethanol before being analyzed.

No ethical approval is required for the experimental methods used in this study.

### Morphological differentiation

The morphological distinction of females of *Cx. pipiens* from other mosquitoes belonging to the genus *Culex* is almost impossible [31]. Thus, only male specimens belonging to the *Cx. pipiens* complex were used to carry out molecular analysis. Being generally accepted that the main diagnostic character of adult mosquitoes is the structure of male genitalia [3], a phallosome analysis was carried out before the genetic analysis in order to identify the samples morphologically. Phallosome was extracted following the protocol described by [32] with some modifications. Mounting of phallosome on microscope slides and further morphological examination of the specimens were made using the identification keys of the Italian Culicidae adults [33]. Observations and measurements were taken with the help of ocular micrometer at 40X of the microscope with phase contrast.

### Genetic analysis

Legs from each mosquito were removed for molecular analysis, while the rest of the body was preserved for future morphological studies.

DNA was extracted according to a previously described salting-out protocol [34]. A region of the mitochondrial DNA corresponding to a fragment of the cytochrome *c* oxidase subunit 1 (COI), tRNA-Leu, and the cytochrome *c* oxidase subunit 2 (COII) was amplified using the universal primer pairs LCO-1490/HCO-2198 [35] and C1-J-2195/TK-N-3796 [36]. Amplifications were performed in 20 μl reactions (1x PCR Go Taq Flexi buffer - Promega, 2.5 mM MgCl_2_, 0.1 mM dNTPs, 0.5 μM for each primer, 0.5 U of Taq polymerase - Promega, 2 μl DNA template). Thermal cycling conditions for the fragment including the 5’ region of the COI were 5 min at 96 °C followed by 4 cycles of 96 °C for 1 min, 47 °C for 1 min, and 72 °C for 1 min, and other 35 cycles of 96 °C for 1 min, 50 °C for 1 min, and 72 °C for 1 min, with a final extension of 72 °C for 5 min; for the fragment including the 5’ region of the COII, PCR conditions were 5 min at 96 °C followed by 35 cycles with a denaturation step of 96 °C for 1 min, an annealing step of 50 °C for 1 min, and an extension step of 72 °C for 1 min, with a final extension of 72 °C for 5 min.

A fragment of the acetylcholinesterase 2 gene (ace-2) was amplified using the primer pairs F1457/B1246 and Acepip/B1246 [23]. The cycling program of the PCR consisted of a first step at 95 °C for 5 min followed by 35 cycles with a denaturation step of 95 °C for 30 s, an annealing step of 60 °C for 30 s, and an extension step of 72 °C for 1 min, with a final extension of 72 °C for 5 min.

PCR products were checked through electrophoresis on 1.0 % agarose gels stained with SYBR® (Invitrogen) and purified using exonuclease and antarctic phosphatase (GE Healthcare) before sequencing.

Sequencing was performed at the BMR Genomics Service (Padova, Italy) on automated DNA sequencers employing for the mitochondrial markers, the primers LCO-1490 and TK-N-3796 and, for the ace-2 fragment, the primers Acepip and B1246.

### Data analysis

Sequences were edited and aligned using MEGA 6.06 [37]. A GenBank BLAST analysis of the sequences obtained was run through the NCBI website (www.ncbi.nlm.nih.gov) to assess the identity of the sequences. For the COI fragment, the integrated bioinformatics platform Barcode of Life Data (BOLD) System database (www.barcodinglife.org) was also used to identify sequences.

A partition homogeneity test was performed for the COI and COII fragments using PAUP* 4.0b10 [38]. Haplotype and nucleotide diversity as well as the pairwise genetic distances between populations using a Kimura 2-parameters model were calculated with Arlequin 3.5 [39]. The presence of population differentiation was also tested and accomplished by conducting exact tests of population differentiation with 100,000 steps in Markov chain, with 10,000 dememorization steps.

Phylogenetic relationships among sequences of the COI-COII data set were estimated using the approximate maximum-likelihood (ML) analysis. A GTR + I + G model implemented in PHYML 2.4.4 software [40] was applied, with neighbor-joining starting tree and 100 bootstrap replications. A haplotype parsimony network of the COI-COII dataset with a probability cut-off at 95 % was reconstructed using the software TCS 1.21 [41].

The demographic history was inferred within each lineage through the Tajima’s *D* test [42] and mismatch distributions of the pairwise genetic differences [43] using Arlequin 3.5. Populations at demographic equilibrium or decreasing in their size should provide significant positive *D* values with a multimodal distribution of pairwise differences, whereas populations that have undergone through a sudden demographic expansion usually show significant negative *D* values with a unimodal distribution [43, 44]. The sudden expansion model was tested through the analysis of the sum of square deviations (SSD) and the raggedness index (r) representing the modality of the distribution obtaining the corresponding *P* values with a parametric bootstrap approach (10,000 replicates).

The ace-2 haplotypes were reconstructed using a Bayesian statistical method implemented in PHASE 2.1 [45, 46]. Two alleles for every mosquito specimen were reported by this software. Individuals with haplotypes that could not be phased with a probability > 90 % were removed from the data set. In addition, we tested for recombination in the remaining dataset using the methods implemented in the RDP4 software [47] with default settings. Moreover, the heterozygote samples bearing haplotypes never found in homozygosis were cloned using the pGEM-T Easy vector (Promega, Madison, WI) following the manufacturer’s protocols with at least 5 clones per reaction, in order to confirm the haplotypes found by PHASE 2.1. Gene and nucleotide diversity were obtained using Arlequin 3.5.

The phylogenetic relationships among the different ace-2 haplotypes were calculated using two methods: the ML and the Bayesian inference methods. The ML tree was obtained following the same methodology reported above for the mitochondrial markers. For the BI analysis of the same data set Mr Bayes 3.1.2 [48] was used. Two independent iterations were run for 5,000,000 generations and sampled every 100 generations. The 50 % majority rule consensus tree and Bayesian posterior probability of support were obtained discarding the first 25 % of sampled generations.

### Links between genetic diversity and landscape features

In order to assess possible correlations between landscape features and genetic diversity for both nuclear and mitochondrial markers, two variables were considered: landscape composition and air distances from the sampling sites to the closest main natural wetlands.

The correlation between genetic diversity and landscape composition was evaluated comparing haplotype and nucleotide diversity with different land cover categories, quantified within a 1,000 m radius, around the sampling site coordinates using detailed land-use maps (Quadro conoscitivo – Regione Veneto -www.regione.veneto.it/web/ambiente-e-territorio/quadro-conoscitivo) in QGIS software (QGIS Development Team, 2014. QGIS Geographic Information System. Open Source Geospatial Foundation Project, http://qgis.osgeo.org).

For each sampling site, we quantified four land cover categories: 1) urban areas, including residential, commercial and industrial areas; 2) rural areas, comprising arable lands with annual crops; 3) tree crops, including poplar plantations and orchards, and 4) wetlands, comprising surface waters and natural wetland areas. The response variables were haplotype and nucleotide diversity, tested separately across the ten sampling sites. The four land cover categories were tested in order to detect possible collinearity between them. The land cover categories that were not correlated were included in the models as explanatory variables.

The correlation between the distances from the sampling sites to the closest main natural wetlands and genetic variability (haplotype and nucleotide diversity) was tested through a regression analysis. We considered as main natural wetlands those with an area at least 10 km^2^. These areas were located along the coast and were included in the Delta area or in the Venice Lagoon. All statistical analyses were carried out using R software [49].

## Results

### Genetic diversity within COI and COII markers

A total of 158 of the 162 adult mosquitoes analyzed, morphologically identified as members of the *Cx. pipiens* complex and representing ten populations (Table 1), were successfully sequenced. A fragment of 571 bp of COI and an additional fragment of 629 bp of the 5’ part of COII gene was obtained for all the individuals. Alignment of sequences showed a total of 19 and 21 polymorphic sites for COI and COII respectively.

All sequences were translated with Transeq (EMBOSS: http://www.ebi.ac.uk/Tools/st/emboss_transeq/) to exclude the presence of nuclear mitochondrial pseudogenes. All of the substitutions, except one, resulted to be synonymous thus not affecting the amino acid sequence. In haplotype 14 of COII fragment, the replacement of a G by an A in pos 22 resulted in the amino acid change G8S. Sequences were deposited in the NCBI database with the GenBank accession numbers reported in Additional file 1: Table S1.

A comparison with GenBank and BoldSystem databases showed for the COI a similarity > 99 % with members of the *Cx. pipiens* complex. In particular, 125 sequences showed a similarity of 100 % with *Cx. p. molestus* [17, 50], two sequences corresponded to *Cx. p. pipiens* [50, 51] while the remaining 31 sequences showed only a 99 % similarity with both sequences of *Cx. pipiens* (undefined form) [16] and sequences of *Cx. quinquefasciatus* [17, 52–54]. On the other hand, COII sequences showed homology only with *Cx. quinquefasciatus* (99–100 %) (i.e. [55]) as for this gene no sequences of *Cx. pipiens* were present in GenBank at the time of writing (July 2015).

Partition homogeneity test confirmed that COI and COII fragments bear a homogeneous signal (*P* = 0.28), allowing data to be pooled for further analyses. Diversity indexes ranged between 0 and 0.92 for the haplotype diversity (*H*) and between 0 and 0.77 % for the nucleotide diversity (*π*). Populations S07 and S08 showed the significantly highest *H* values (*H* = 0.92 and 0.6 respectively) while S02 and S05 showed the lowest (*H* = 0). Regarding nucleotide diversity, population S07 displayed the highest value together with populations S06, S08 and S09 while populations S02, S05 and S10 showed the lowest variability. The distribution of haplotypes among the ten populations and other summary statistics are shown in Table 2.

**Table 2:** Descriptive statistics of mitochondrial and nuclear DNA markers, with the number of individuals analyzed (N)

	COI-COII				ace-2		
Population	N	Haplotypes	*H*	*π*(%)	N	Gd	*π*(%)
S01	13	A1(12), F7(1)	0.15	0.11	4	0.61	0.41
S02	9	A1(9)	0.00	0.00	4	0.82	0.39
S03	19	A1(17), P14(2)	0.20	0.20	5	0.82	0.42
S04	17	A1(14), B2(1), C6(1), G8(1)	0.33	0.20	7	0.69	0.35
S05	8	A1(8)	0.00	0.00	-	-	-
S06	22	A1(16), H4(2), J7(1), L20(1), R19(2)	0.47	0.48	10	0.86	0.52
S07	9	A1(3), D12(1), G7(1), G9(1), I7(1), K9(1), Q15(1)	0.92	0.77	6	0.92	0.64
S08	22	A1(14), D13(1), G10(1), G11(1), H3(1), M18(1), O16(2), S19(1)	0.60	0.59	8	0.78	0.39
S09	25	A1(19), E3(1), E5(1), G7(1), G10(1), K7(1), N17(1)	0.43	0.34	9	0.71	0.33
S10	14	A1(13), B1(1)	0.14	0.01	9	0.64	0.42

For each population all the haplotypes found are reported. In brackets the number of individuals bearing each haplotype. *H*: haplotype diversity, *π* (%): nucleotide diversity, Gd: gene diversity

Pairwise genetic distances (*F*_*st*_) between analyzed populations showed significant *F*_*st*_ values only for population S07 when compared to all the other populations except for populations S06 and S08 (Table 3). On the other hand, exact test of population differentiation was significant (*P* < 0.05) for all the pairwise comparisons, indicating a non-random distribution of haplotypes among populations.

**Table 3:** Pairwise genetic distances (*F*_*st*_) between populations obtained from the mitochondrial data set

Population	N	S01	S02	S03	S04	S05	S06	S07	S08	S09
S01	13									
S02	9	-0.031								
S03	19	0.005	-0.001							
S04	17	-0.044	-0.014	0.007						
S05	8	-0.042	0.000	-0.012	-0.024					
S06	22	0.021	0.043	0.023	0.018	0.033				
S07	9	0.304^a^	0.357^a^	0.331^a^	0.286^a^	0.336^a^	0.126			
S08	22	0.059	0.082	0.051	0.053	0.071	-0.034	0.075		
S09	25	-0.003	0.048	0.047	-0.000	0.038	-0.010	0.151^a^	0.009	
S10	14	-0.010	-0.035	0.023	0.003	-0.045	0.070	0.424^a^	0.111	0.071

*P* < 0.01 were considered significant (^a^)

### Relationships between mitochondrial haplotypes

**Fig. 1:**
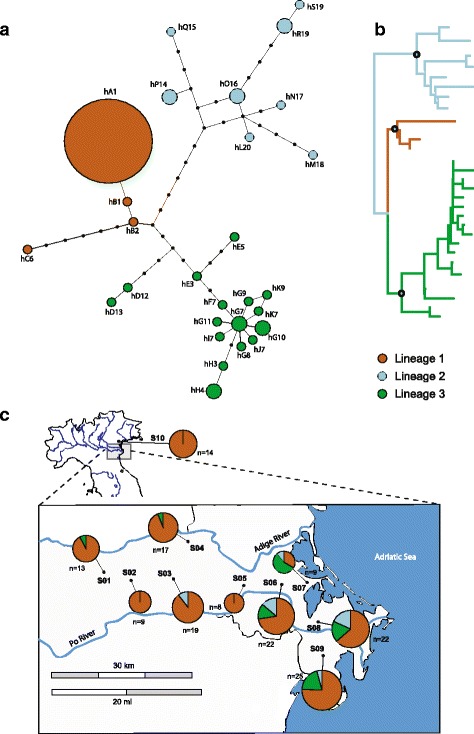
Phylogenetic reconstructions of the *Culex pipiens* complex based on the mitochondrial dataset (COI and COII) and geographical distribution. **a** Haplotypes network realized by TCS 1.21. Each haplotype is represented by a circle, with the area of the circle proportional to its frequency. Numbers denote haplotype identifiers presented in Additional file 3: Table S2. Each line represents a single mutation while small black dots symbolize intermediate missing or unsampled haplotype. The color represents the mitochondrial lineages previously identified by the maximum-likelihood phylogenetic tree; **b** Summarized maximum-likelihood phylogenetic tree haplotypes included in the analysis; branch supports are reported as ML bootstraps in figure 1S; **c** Map showing the proportional geographic distribution of the mtDNA *Cx. pipiens* complex lineages across sampled populations. Letters indicate the location code, reported in Table 1; ‘n’ indicates the sample size

A statistical parsimony network obtained joining COI and COII sequences included 28 haplotypes. Three major lineages were identified and reported in the haplotype network (Fig. 1a) through bootstrap supports calculated on an ML tree (Fig. 1b and Additional file 2: Figure S1). Lineage 1 consisted in four haplotypes including three unique haplotypes (B1, B2, and C6) and the most frequent haplotype (A1) present in approximately 79 % (*n* = 125) of the mosquitoes analyzed. This haplotype was found in all the populations analyzed and in particular it was exclusive in populations S02 and S05 (Table 2; Fig. 1c). Lineage 2 grouped together eight rare haplotypes mainly present in the populations close to the Delta area (S03, S06, S07, S08, and S09) (Fig. 1c). This clade was separated from lineage 1 by nine mutational steps with missing intermediate haplotypes. Moreover, within the clade, seven haplotypes were separated from each other by several missing intermediate haplotypes (i.e. 11 mutational steps separated the haplotype Q15 from M18). Lineage 3 included 16 rare haplotypes with 12 of them connected in a star-shape manner with an inner haplotype (G7) shared by only two samples. Only three of the haplotypes were represented by two sequences each, while the remaining 13 were represented by a single sequence. The haplotypes of this clade were present at higher frequency in populations of the Delta area (S06, S07, S08 and S09). Interestingly, more than 50 % of the samples of population S07 bore haplotypes of this clade (Fig. 1c).

### Demographic history

Tajima’s *D* test was applied in each of the three lineages identified in order to check for past demographic events. The null hypothesis of neutrality was rejected only in lineage 1 (*D* = -2.07; *P* < 0.01) suggesting a past population expansion.

**Fig. 2:**
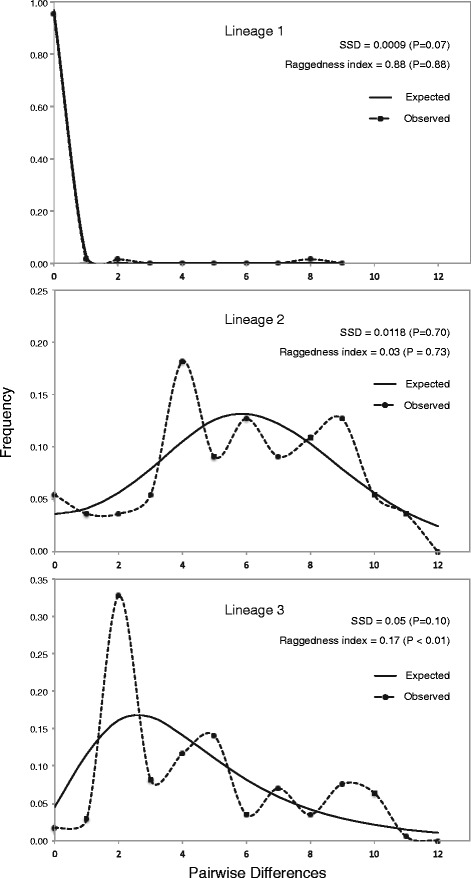
Mismatch distribution under population expansion model of the three mitochondrial lineages based on pairwise differences. The sums of squared deviations (SSD), raggedness index (r) and their corresponding *P-*values are given

The analysis of mismatch distributions for the three lineages revealed differences among their demographic histories. Mismatch distribution of lineage 1 was unimodal and characterized by small observed mean (0.17 ± 0.01 S.E.M.). In contrast, the mismatch distributions in lineage 2 and lineage 3 were broadly multimodal, with higher mismatch observed means of 5.82 ± 0.39 S.E.M and 4.47 ± 0.21 S.E.M, respectively as expected under a model of relative constant population size (Fig. 2). Moreover, lineage 1 and lineage 2 had SSD and raggedness index values that did not reject a sudden expansion model. Conversely, lineage 3, although not significant for the SSD, showed a significant raggedness index (*r* = 0.17, *P* < 0.01) suggesting that this lineage may have had a relatively stable population size over time.

### Genetic diversity within ace-2 marker region

A subset of 74 individuals of the 158 mosquitoes previously sequenced for the mitochondrial DNA was used for the nuclear analysis. In particular, all the samples showing rare mitochondrial haplotypes and a representative part of the samples with the most frequent haplotype (A1) in the combined data set (COI-COII) were chosen for these analyses. Sequencing of the ace-2 gene yielded a 502 bp fragment with the exon 3 spanning between positions 313–502. Through the analysis carried out with the software PHASE, 12 samples were removed since their alleles could not be correctly assigned (*P* < 90 %). Thus a total of 16 unique alleles of ace-2 gene were identified in the remaining 62 samples. Cloning confirmed the haplotypes found by PHASE whereas the RDP4 software did not detect any statistically significant recombinant among the ace-2 haplotypes. Variable sites were mainly found in intron 2 (15 polymorphic sites out of 312 bp) while only one nucleotide substitution out of 190 bp was found in exon 3, that did not change the inferred amino acid sequence. The sequences were deposited in the NCBI database with the GenBank accession numbers reported in Additional file 1: Table S1. A BLAST analysis of these sequences showed a similarity between 99–100 % with *Cx. pipiens* [5, 24].

Gene diversity related to ace-2 was high in all the populations analyzed, with values ranging between 0.61 (population S01) and 0.92 (populations S07). Nucleotide diversity (*π*) showed values between 0.33 and 0.42 for all the populations except for populations S06 and S07, which showed the highest values (*π* = 0.52 % and *π* = 0.64 %, respectively) (Table 2).

**Fig. 3:**
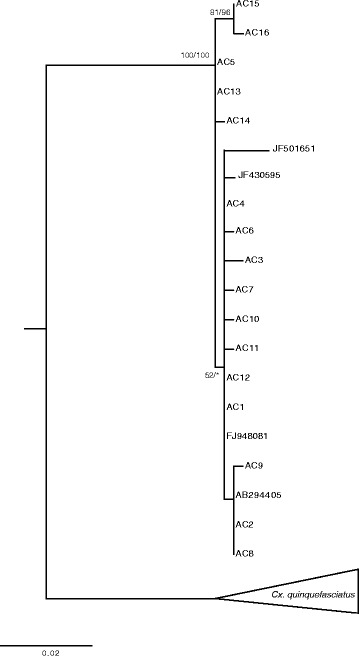
Phylogenetic tree of *Culex pipiens* complex based on ace-2 marker. The tree was reconstructed using the nucleotide sequence alignment of 502 nucleotides from 62 field collected mosquitoes. The first number on each branch indicates the value of bootstrap probability (Bp) from the bootstrap test of the ML analysis and the second number indicates the value of posterior probability (Pp) from the Bayesian analysis. Bp values >70 % and Pp values >95 % were considered statistically significant [82, 83]. Sequences JF430595, JF501651, FJ948081 (*Cx. pipiens* form pipiens) and the sequence AB294405 (*Cx. pipiens* form molestus) were retrieved from GenBank. The collapsed cluster groups *Cx. quinquefasciatus* sequences retrieved in GenBank with the following accession numbers: FJ210901, GQ165791-GQ165798, FN395201-FN395202, FN395204-FN395205, FJ210909-FJ210910, GU188856, HQ398883

Phylogenetic analysis conducted on the ace-2 alleles found in this study (Additional file 3: Table S2), showed that all the alleles were grouped in one highly supported cluster (Bp/Pp = 100/100) including the ace-2 haplotypes retrieved from GenBank corresponding to *Cx. p. pipiens* [FJ948081, JF430595, JF501651] and *Cx. p. molestus* [AB294405] (Fig. 3). No internal structure was present within this cluster with the exception of a subclade grouping two haplotypes supported by both maximum likelihood and Bayesian inference (Bp/Pp = 81/97).

### Links between genetic diversity and landscape features

The landscape composition quantified within a 1,000 m radius around on the sampling site coordinates showed a high percentage of urban and rural areas in all the sites, with an average percentage of 19 and 66 % respectively. The other two categories (tree crops and wetlands) presented a low coverage in almost all the sites, with the exception of S07 and S08 where a high percentage of the latter category was observed (42 and 33 %, respectively) (Table 1).

Since an initial analysis showed a significant inverse correlation between rural and urban land categories only the latter was included in the models. The analyses for the mitochondrial diversity showed that only wetland areas category was significantly and positively correlated with *Cx. pipiens* complex genetic diversity, for both the nucleotide (*P* < 0.01) and haplotype (*P* = 0.0 < 1) diversity. Conversely, no significant correlations were found when considering the nuclear marker diversity.

**Fig. 4:**
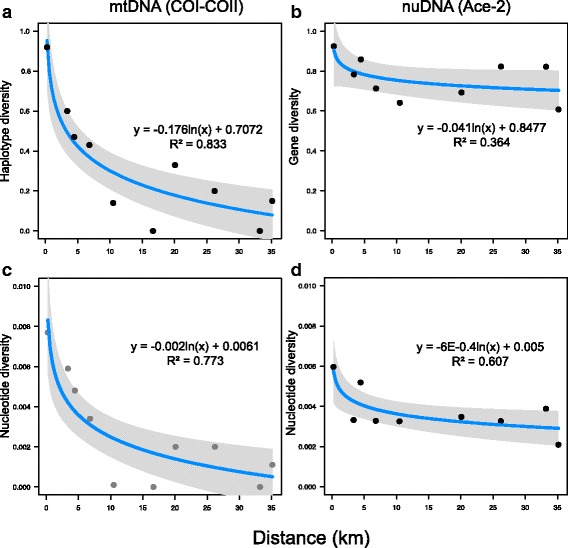
Relationship between genetic diversity and geographical distances from sampling sites to the main wetland areas. **a** haplotype and **c** nucleotide diversity of COI-COII against geographical distance; **b** gene and **d** nucleotide diversity of the ace-2 gene against geographical distance

Finally, we found a significant inverse effect of the distance from the sampling sites to the main natural wetland areas on the mitochondrial haplotype (R^2^ = 0.83; *P* < 0.001) and nucleotide (R^2^ = 0.77; *P* < 0.001) diversity of each population, that decrease from the main natural wetlands close to the coast to the inner areas in the mainland (Fig. 4a-c). On the other hand, nuclear marker diversity did not show any significant correlation with the distance from the main natural wetland areas (Fig. 4b-d).

## Discussion

*Cx. pipiens* populations in the WNV outbreak area along the last part of the Po River showed a high genetic diversity in both mitochondrial and nuclear markers. In particular, for the COI and COII gene fragments, the diversity was mainly concentrated in four populations occurring in the lowlands in the Delta area (S06, S07, S08, S09), with one population (S07) showing also a significant genetic distance from the other populations. A different pattern of variability was found in the ace-2 marker, with all the populations showing homogenous genetic variability values with population S07 displaying the highest nucleotide diversity value (Table 3). This outcome is even more interesting considering that in this study we examined the population genetic structure across a small geographical area.

The high variability found in the Delta area, with the presence of several rare haplotypes, contrasts with the low genetic diversity found in other areas in previous studies. A lowest genetic variability for COI was found for example in most of the populations analyzed in Germany [16, 56] and Russia [57]. Similarly, a low intraspecific variability has been observed for the COII in populations collected around the world and reared in laboratory [58]. This difference in genetic variability could be due to the fact that most of these surveys on the *Cx. pipiens* complex have been mainly focused on urban and suburban environments and do not include natural habitats. In general, surveillance efforts for potential disease-carrying mosquitoes have necessarily focused on high human population density areas with a greater risk of disease transmission to human populations [59].

The haplotypes found in this study are structured in three distinct lineages showing differences in their composition (Fig. 1a). Interestingly, the high genetic variability was condensed into two lineages (lineage 2 and 3) mainly linked to the populations of the Delta area. These two lineages were characterized by the presence of several rare haplotypes without any high frequency haplotype. This pattern is consistent with what is expected for populations that have not experienced past demographic expansions as highlighted by both the neutrality test and the multimodality of the mismatch distribution curves of these two lineages [44]. Moreover, clues of past bottlenecks may be inferred for lineage 2 due to the presence of many peaks at larger values of pairwise nucleotide differences, usually generated after drastic contractions of population size [43, 60]. Due to the fine-scale sampling of this study it is premature to speculate on the causes that led to these past demographic events in the two lineages and further phylogeographic analyses would be needed extending the research on a wider range. In contrast with the high variability of these two lineages a reduced mitochondrial variability was found in lineage 1. This lineage was characterized by the presence of one haplotype shared by high number of individuals and only other three rare haplotypes. This may be related to a recent population expansion as suggested by both the neutrality test and the unimodal mismatch distribution. Interestingly, the most common haplotype found in our sampling and belonging to the lineage 1, is reported to be associated with *Cx. pipiens* form molestus, mainly linked to human-made environments [50, 61]. The presence of a high frequency haplotype could be also the result of selective sweeps due to symbiotic bacteria. Possible influence of *Wolbachia* producing selective sweeps has been suggested by several authors in order to justify the reduced haplotype diversity in *Cx. pipiens* [17, 22, 58] and *Cx. quinquefasciatus* [62, 63].

The occurrence of three mitochondrial lineages may underlie the existence of cryptic species within *Cx. pipiens* as it has already been suggested for the strong genetic structure found in *Cx. torrentium* [56]. Noteworthy, some of our haplotypes of lineages 2 and 3 resulted similar to haplotypes retrieved from GenBank of the southern house mosquito, *Cx. quinquefasciatus*. However, the morphological analysis excluded that the specimens with these haplotypes could belong to *Cx. quinquefasciatus* as they presented the divergent dorsal arms of the male genitalia characteristic of *Cx. pipiens* in contrast to the parallel dorsal arms of *Cx. quinquefasciatus* [15]. Moreover, sequences obtained for the ace-2, a marker successfully used to distinguish the *Cx. pipiens* and *Cx. quinquefasciatus* [23], confirmed the morphological results as they showed a strong differentiation from the *Cx. quinquefasciatus* sequences deposited in GenBank (Fig. 3).

The high variability found for ace-2 in the populations of *Cx. pipiens* analyzed is consistent with the diversity found in other studies for this nuclear marker [64, 65] and is largely due to the higher mutation rate of the intron region [66–68]. On the other hand, in contrast to the well differentiated mitochondrial haplotype structure, all the ace-2 sequences grouped in a supported cluster without a strong internal subdivision.

Several studies report discordant patterns between mtDNA and nuclear markers in animals [69]. These incongruences could be explained by diverse factors such as differences in effective population sizes between the two markers, sex-biased dispersal and selection. Discordances between mitochondrial and nuclear markers are expected as mitochondrial genome is haploid and usually uniparentally inherited leading to a fourfold smaller effective population size [70, 71]. Mitochondrial DNA can thus complete the process of lineage sorting faster than nuclear DNA, resulting in genetic differences between populations, whereas nuclear markers do not reflect yet this differentiation due to their longer coalescence times. The discordance between the two markers used could be also the result of a sex-biased dispersal with males spreading on a wider range than females, leading to nuclear gene flow between populations without a corresponding mitochondrial gene flow. A lower dispersal potential of females has been observed in other mosquitoes, such as in *Cx. tarsalis* [72], *Ochlerotatus* sp. [73] and *Anopheles* sp. [74] hypothesizing that mosquitoes may have a strong home range memory and tend to return to their natal breeding sites for ovipositing and blood-feeding [75, 76]. Another factor that is rarely considered but could be potentially relevant, is selection. Environmental characteristics, such as spatial variation in habitat quality, could favor one mitochondrial variant over another. These variants could be associated with different biological or behavioral features of individuals [77, 78]. In our case, we may assume that mosquito females dispersing outside their own habitat may show a lower fitness than local females, due to different behaviors and ecological requirements (e.g. host choice, mating behavior and oviposition site choice).

Understanding landscape effects on genetic structure could provide insight into biological processes such as metapopulation dynamics and species distribution [79]. In our study, a significant effect of landscape composition on genetic diversity was found, with positive correlation between mitochondrial variability and percentage of wetland areas. An even more significant effect was found considering the distance from the main wetland areas to sampling sites, with the mitochondrial genetic variability decreasing from the main wetland areas along the coast to the inner areas (Fig. 4a-c). Large patches of natural wetlands could maintain higher genetic diversity serving as a source of variability for smaller natural patches, suggesting a model of population expansion between the mainland-island and the source-sink model [80]. Natural environments could be more suitable to harbour populations with higher genetic variability due to the presence of more undisturbed and stable breeding sites sustaining permanent mosquito populations. In addition, in this natural and scarcely urbanized context, where the insecticide treatments are low or absent [81], mosquito populations may undergo to lower selective pressure thus being less prone to bottlenecks. On the other hand, the low genetic differentiation found in inner and more anthropized sites could also be explained by a chemical control of mosquito populations in these environments. A low genetic differentiation induced by insecticide-resistance has been suggested within the genus *Culex* [16, 55]. In anthropized areas, the mosquitoes may have experienced population bottlenecks caused by programs of vector-borne disease control (i.e. larviciding, fogging and elimination of breeding sites) [81].

## Conclusion

The Delta Po River is on the route of migratory birds that play an important role in the introduction of viruses, such as WNV, into Europe and the Mediterranean Basin [6]. Here, a high genetic variability of the *Cx. pipiens* complex, one of the primary WNV vectors, was found using both mitochondrial and nuclear markers. In particular, the mitochondrial diversity was positively correlated to the natural wetland areas close to the Delta Po River. The presence of different haplotypes in the *Cx. pipiens* complex populations of this area could underlie differences in their biology as it has been already observed for the forms pipiens and molestus [18, 19] that show different feeding behaviors. In this scenario, the cryptic diversity found in the Po River Delta is more interesting as we cannot completely exclude a linkage between the three mitochondrial lineages found and the biology of the mosquitoes and hence their vector ability of arboviral infections, complicating in this way the arbovirus transmission network. Since the link between WNV and deltaic areas seems clear [7], we think that it should be important to further extend genetic studies of the *Cx. pipiens* complex in delta areas and other natural habitats. In addition, these studies could be coupled with vector competence studies, because of the potential risk of the different forms as vectors of arbovirus.

## Additional files

Additional file 1: Table S1. GenBank accession numbers for the haplotypes found. (DOCX 18 kb) Additional file 2: Figure S1. Phylogenetic tree of *Culex pipiens* complex based on mitochondrial COI and COII markers. (PDF 286 kb) Additional file 3: Table S2. Summary of *Culex pipiens* complex samples analyzed with reference to individual haplotypes. (DOCX 50 kb)

## Abbreviations

ace-2: acetylcholinesterase-2 gene
BI: bayesian inference
BLAST: Basic Local Alignment Search Tool
BOLD: Barcode of Life Data Systems
Bp: bootstrap probability
COI: cytochrome oxidase subunit 1
COII: cytochrome oxidase subunit 2
DNA: deoxyribonucleic acid
dNTPs: deoxynucleotide triphosphates
*F*_*st*_: fixation index
G8S: Glycine to Serine in position 8
Gd: genetic diversity
GTR + I + G: General Time Reversible + Invariant + Gamma
*H*: haplotype diversity
ML: maximum likelihood
*P*: probability
PCR: polymerase chain reaction
pos: position
Pp: posterior probability
r: raggedness index
R^2^: coefficient of determination
S.E.M.: standard error of the mean
SSD: sums of squared deviations
WNV: West Nile virus
*π*: nucleotide diversity
