# Supplementary material for: High genetic diversity in the Culex pipiens complex from a West Nile Virus epidemic area in Southern Europe
Source: Parasit Vectors. 2016 Mar 15;9:150. doi: 10.1186/s13071-016-1429-1 (PMC4791856; doi:10.1186/s13071-016-1429-1)
Supplement: Additional file 1: Table S1. — GenBank accession numbers for the haplotypes found. (DOCX 18 kb) [file 13071_2016_1429_MOESM1_ESM.docx]

**Table S1**

| COI haplotype | A | KM922633 |
| --- | --- | --- |
|  | B | KM922634 |
|  | C | KM922642 |
|  | D | KM922644 |
|  | E | KM922648 |
|  | F | KM922641 |
|  | G | KM922640 |
|  | H | KM922636 |
|  | I | KM922632 |
|  | J | KM922645 |
|  | K | KM922649 |
|  | L | KM922646 |
|  | M | KM922638 |
|  | N | KM922647 |
|  | O | KM922637 |
|  | P | KM922643 |
|  | Q | KM922650 |
|  | R | KM922635 |
|  | S | KM922639 |
| COII haplotype | 1 | KM922652 |
|  | 2 | KM922662 |
|  | 3 | KM922669 |
|  | 4 | KM922656 |
|  | 5 | KM922651 |
|  | 6 | KM922660 |
|  | 7 | KM922654 |
|  | 8 | KM922663 |
|  | 9 | KM922667 |
|  | 10 | KM922665 |
|  | 11 | KM922659 |
|  | 12 | KM922668 |
|  | 13 | KM922664 |
|  | 14 | KM922661 |
|  | 15 | KM922671 |
|  | 16 | KM922657 |
|  | 17 | KM922670 |
|  | 18 | KM922658 |
|  | 19 | KM922655 |
|  | 20 | KM922666 |
| ace-2 allele | AC1 | KM922614 |
|  | AC2 | KM922603 |
|  | AC3 | KM922618 |
|  | AC4 | KM922622 |
|  | AC5 | KM922631 |
|  | AC6 | KM922617 |
|  | AC7 | KM922615 |
|  | AC8 | KM922602 |
|  | AC9 | KM922600 |
|  | AC10 | KM922616 |
|  | AC11 | KM922624 |
|  | AC12 | KM922628 |
|  | AC13 | KM922629 |
|  | AC14 | KM922601 |
|  | AC15 | KM922611 |
|  | AC16 | KM922630 |
|  |  |  |
