# Supplementary figures and images for: High genetic diversity in the Culex pipiens complex from a West Nile Virus epidemic area in Southern Europe
Source: Parasit Vectors. 2016 Mar 15;9:150. doi: 10.1186/s13071-016-1429-1 (PMC4791856; doi:10.1186/s13071-016-1429-1)

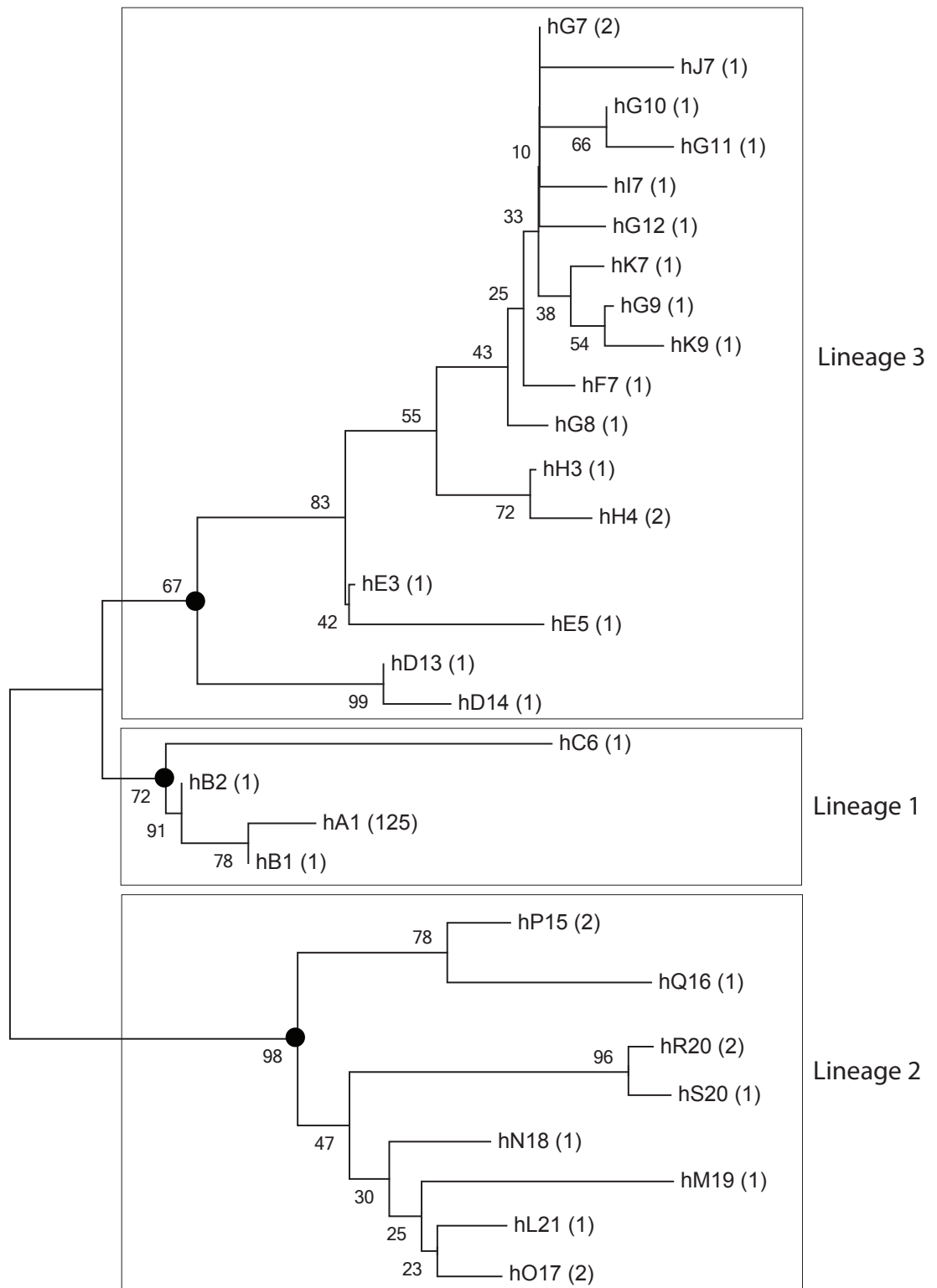

Supplement: Additional file 2: Figure S1. — Phylogenetic tree of Culex pipiens complex based on mitochondrial COI and COII markers. (PDF 286 kb) [file 13071_2016_1429_MOESM2_ESM.pdf]
