# Supplementary material for: High genetic diversity in the Culex pipiens complex from a West Nile Virus epidemic area in Southern Europe
Source: Parasit Vectors. 2016 Mar 15;9:150. doi: 10.1186/s13071-016-1429-1 (PMC4791856; doi:10.1186/s13071-016-1429-1)
Supplement: Additional file 3: Table S2. — Summary of Culex pipiens complex samples analyzed with reference to individual haplotypes. (DOCX 50 kb) [file 13071_2016_1429_MOESM3_ESM.docx]

**Table S2**

| **ID**  **sample** | **Study**  **site** | **COI**  **haplotype** | **COII**  **haplotype** | **Combined**  **COI & COII haplotype** | **ace-2**  **haplotype by PHASE** |
| --- | --- | --- | --- | --- | --- |
| 3 | S04 | A | 1 | A1 |  |
| 7 | S03 | A | 1 | A1 |  |
| 8 | S03 | A | 1 | A1 |  |
| 9 | S03 | A | 1 | A1 | AC2, AC4 |
| 10 | S03 | A | 1 | A1 |  |
| 12 | S04 | A | 1 | A1 | AC1, AC1 |
| 13 | S04 | A | 1 | A1 |  |
| 14 | S04 | A | 1 | A1 | AC1, AC6 |
| 15 | S03 | A | 1 | A1 |  |
| 16 | S03 | A | 1 | A1 | AC1, AC4 |
| 17 | S03 | P | 14 | P14 | AC1, AC3 |
| 18 | S03 | A | 1 | A1 | AC1, AC14 |
| 19 | S04 | A | 1 | A1 |  |
| 20 | S04 | A | 1 | A1 | AC1, AC8 |
| 21 | S04 | A | 1 | A1 |  |
| 22 | S04 | A | 1 | A1 |  |
| 23 | S03 | A | 1 | A1 |  |
| 24 | S03 | A | 1 | A1 |  |
| 25 | S03 | A | 1 | A1 |  |
| 26 | S03 | A | 1 | A1 |  |
| 27 | S04 | G | 8 | G8 | AC4, AC8 |
| 28 | S04 | A | 1 | A1 |  |
| 29 | S04 | B | 2 | B2 | AC1, AC8 |
| 30 | S04 | A | 1 | A1 |  |
| 31 | S03 | A | 1 | A1 |  |
| 32 | S03 | P | 14 | P14 | AC1, AC2 |
| 33 | S03 | A | 1 | A1 |  |
| 34 | S03 | A | 1 | A1 |  |
| 35 | S04 | A | 1 | A1 |  |
| 36 | S04 | A | 1 | A1 |  |
| 37 | S04 | A | 1 | A1 |  |
| 38 | S03 | A | 1 | A1 |  |
| 39 | S03 | A | 1 | A1 |  |
| 40 | S03 | A | 1 | A1 |  |
| 41 | S04 | C | 6 | C6 | AC1, AC1 |
| 42 | S04 | A | 1 | A1 | AC4, AC8 |
| 43 | S01 | A | 1 | A1 |  |
| 44 | S01 | A | 1 | A1 | AC1, AC1 |
| 45 | S01 | A | 1 | A1 |  |
| 46 | S01 | A | 1 | A1 |  |
| 47 | S01 | A | 1 | A1 |  |
| 48 | S01 | A | 1 | A1 |  |
| 49 | S01 | A | 1 | A1 |  |
| 50 | S01 | A | 1 | A1 |  |
| 51 | S01 | A | 1 | A1 |  |
| 52 | S01 | A | 1 | A1 | AC1, AC1 |
| 53 | S02 | A | 1 | A1 |  |
| 54 | S02 | A | 1 | A1 |  |
| 55 | S02 | A | 1 | A1 | AC1, AC2 |
| 56 | S02 | A | 1 | A1 | AC1, AC8 |
| 57 | S02 | A | 1 | A1 |  |
| 58 | S02 | A | 1 | A1 |  |
| 59 | S02 | A | 1 | A1 |  |
| 60 | S02 | A | 1 | A1 | AC1, AC11 |
| 61 | S02 | A | 1 | A1 | AC2, AC8 |
| 62 | S01 | A | 1 | A1 | AC2, AC8 |
| 63 | S01 | A | 1 | A1 |  |
| 64 | S01 | F | 7 | F7 | AC1, AC2 |
| 84 | S09 | A | 1 | A1 |  |
| 85 | S09 | A | 1 | A1 |  |
| 86 | S09 | A | 1 | A1 | AC1, AC8 |
| 87 | S08 | A | 1 | A1 | AC1, AC7 |
| 88 | S08 | G | 11 | G11 | AC2, AC3 |
| 89 | S08 | S | 19 | S19 | AC1, AC3 |
| 90 | S08 | A | 1 | A1 |  |
| 91 | S08 | M | 18 | M18 | AC1, AC3 |
| 92 | S08 | O | 16 | O16 | AC3, AC11 |
| 93 | S06 | A | 1 | A1 | AC2, AC9 |
| 94 | S06 | A | 1 | A1 |  |
| 95 | S06 | H | 4 | H4 | AC3, AC16 |
| 96 | S06 | R | 19 | R19 | AC4, AC13 |
| 97 | S06 | R | 19 | R19 | AC1, AC1 |
| 98 | S06 | A | 1 | A1 | AC1, AC8 |
| 99 | S07 | I | 7 | I7 | AC3, AC5 |
| 100 | S07 | G | 7 | G8 | AC1, AC11 |
| 101 | S07 | K | 9 | K9 | AC1, AC3 |
| 102 | S07 | Q | 15 | Q15 | AC2, AC16 |
| 104 | S09 | A | 1 | A1 |  |
| 105 | S10 | A | 1 | A1 |  |
| 106 | S10 | B | 1 | B1 | AC1, AC1 |
| 107 | S10 | A | 1 | A1 | AC1, AC1 |
| 108 | S09 | A | 1 | A1 |  |
| 109 | S09 | A | 1 | A1 |  |
| 110 | S09 | G | 7 | G7 |  |
| 111 | S09 | A | 1 | A1 | AC1, AC8 |
| 112 | S09 | A | 1 | A1 |  |
| 113 | S09 | K | 7 | K7 | AC1, AC3 |
| 114 | S09 | E | 3 | E3 | AC3, AC4 |
| 115 | S09 | A | 1 | A1 |  |
| 116 | S09 | N | 17 | N17 | AC1, AC3 |
| 117 | S08 | A | 1 | A1 |  |
| 118 | S08 | A | 1 | A1 |  |
| 119 | S08 | A | 1 | A1 |  |
| 121 | S06 | A | 1 | A1 |  |
| 122 | S06 | A | 1 | A1 |  |
| 123 | S06 | A | 1 | A1 |  |
| 124 | S06 | A | 1 | A1 | AC8, AC8 |
| 125 | S06 | A | 1 | A1 |  |
| 126 | S08 | A | 1 | A1 |  |
| 127 | S08 | A | 1 | A1 |  |
| 128 | S08 | A | 1 | A1 |  |
| 129 | S08 | H | 3 | H3 | AC1, AC3 |
| 130 | S09 | A | 1 | A1 |  |
| 132 | S05 | A | 1 | A1 |  |
| 133 | S05 | A | 1 | A1 |  |
| 134 | S05 | A | 1 | A1 |  |
| 135 | S05 | A | 1 | A1 |  |
| 136 | S05 | A | 1 | A1 |  |
| 137 | S10 | A | 1 | A1 | AC14, AC15 |
| 138 | S10 | A | 1 | A1 | AC1, AC9 |
| 139 | S10 | A | 1 | A1 | AC1, AC5 |
| 140 | S10 | A | 1 | A1 |  |
| 141 | S07 | A | 1 | A1 |  |
| 142 | S07 | A | 1 | A1 |  |
| 143 | S07 | D | 12 | D12 | AC1, AC12 |
| 144 | S07 | G | 9 | G9 |  |
| 145 | S07 | A | 1 | A1 | AC8, AC8 |
| 146 | S06 | A | 1 | A1 |  |
| 147 | S05 | A | 1 | A1 |  |
| 148 | S05 | A | 1 | A1 |  |
| 149 | S05 | A | 1 | A1 |  |
| 150 | S06 | A | 1 | A1 |  |
| 151 | S06 | A | 1 | A1 | AC1, AC15 |
| 152 | S06 | A | 1 | A1 |  |
| 153 | S06 | H | 4 | H4 | AC1, AC3 |
| 154 | S06 | A | 1 | A1 |  |
| 155 | S06 | A | 1 | A1 |  |
| 156 | S09 | A | 1 | A1 |  |
| 157 | S09 | A | 1 | A1 |  |
| 158 | S09 | A | 1 | A1 |  |
| 159 | S09 | A | 1 | A1 |  |
| 160 | S09 | A | 1 | A1 |  |
| 161 | S06 | L | 20 | L20 | AC1, AC10 |
| 162 | S06 | J | 7 | J7 | AC1, AC3 |
| 163 | S06 | A | 1 | A1 |  |
| 164 | S06 | A | 1 | A1 |  |
| 165 | S08 | A | 1 | A1 |  |
| 166 | S08 | G | 10 | G10 |  |
| 167 | S08 | A | 1 | A1 | AC1, AC2 |
| 168 | S08 | O | 16 | O16 | AC1, AC5 |
| 169 | S08 | D | 13 | D13 |  |
| 170 | S08 | A | 1 | A1 |  |
| 171 | S08 | A | 1 | A1 |  |
| 172 | S08 | A | 1 | A1 |  |
| 174 | S08 | A | 1 | A1 |  |
| 175 | S09 | A | 1 | A1 |  |
| 176 | S10 | A | 1 | A1 | AC1, AC2 |
| 177 | S10 | A | 1 | A1 |  |
| 178 | S10 | A | 1 | A1 |  |
| 179 | S10 | A | 1 | A1 | AC1, AC1 |
| 180 | S10 | A | 1 | A1 |  |
| 181 | S10 | A | 1 | A1 | AC1, AC8 |
| 182 | S10 | A | 1 | A1 | AC1, AC12 |
| 183 | S09 | A | 1 | A1 |  |
| 184 | S09 | G | 10 | G10 | AC1, AC3 |
| 186 | S09 | A | 1 | A1 | AC1, AC1 |
| 188 | S09 | E | 5 | E5 | AC1, AC1 |
| 190 | S09 | A | 1 | A1 | AC2, AC2 |
